# Earth's anomalous middle-age magmatism driven by plate slowdown

**DOI:** 10.1038/s41598-022-13885-9

**Published:** 2022-06-21

**Authors:** C. O’Neill, M. Brown, B. Schaefer, J. A. Gazi

**Affiliations:** 1 Department of Earth and Environmental Science, Macquarie Planetary Research Centre, Macquarie University, Sydney, 2109 Australia; 2 Laboratory for Crustal Petrology, Department of Geology, University of Maryland, College Park, MD 20742-4211 USA; 3 Present Address: Origins Research Institute (ORI), Research Centre for Astronomy and Earth Sciences, 15-17 Konkoly Thege Miklós Road, Budapest, 1121 Hungary

**Keywords:** Geodynamics, Precambrian geology, Geophysics, Tectonics

## Abstract

The mid-Proterozoic or "boring billion" exhibited extremely stable environmental conditions, with little change in atmospheric oxygen levels, and mildly oxygenated shallow oceans. A limited number of passive margins with extremely long lifespans are observed from this time, suggesting that subdued tectonic activity—a plate slowdown—was the underlying reason for the environmental stability. However, the Proterozoic also has a unique magmatic and metamorphic record; massif-type anorthosites and anorogenic Rapakivi granites are largely confined to this period and the temperature/pressure (thermobaric ratio) of granulite facies metamorphism peaked at over 1500 °C/GPa during the Mesoproterozoic. Here, we develop a method of calculating plate velocities from the passive margin record, benchmarked against Phanerozoic tectonic velocities. We then extend this approach to geological observations from the Proterozoic, and provide the first quantitative constraints on Proterozoic plate velocities that substantiate the postulated slowdown. Using mantle evolution models, we calculate the consequences of this slowdown for mantle temperatures, magmatic regimes and metamorphic conditions in the crust. We show that higher mantle temperatures in the Proterozoic would have resulted in a larger proportion of intrusive magmatism, with mantle-derived melts emplaced at the Moho or into the lower crust, enabling the production of anorthosites and Rapakivi granites, and giving rise to extreme thermobaric ratios of crustal metamorphism when plate velocities were slowest.

## Introduction

Fluctuations in global plate velocities are well documented for the Phanerozoic^1^, and have significant effects on global volcanic and climate systems. Even larger variations in the global average have been predicted for the Proterozoic by earlier models^2^, and plate slowdowns are implicated in observed tectonomagmatic lulls^3,4^. While direct constraints on Precambrian plate velocities are limited^2,5,6^, a proxy exists in the form of passive margin distributions through time; in the Mesoproterozoic (1.6–1.0 Ga) these margins demonstrate extreme longevity, peaking at ca. 1500 Ma with lifespans around 600 Myr^7^. As passive margin lifetimes are governed by the Wilson cycle, they record the tempo of tectonic activity, and the Mesoproterozoic peak in lifespans has been suggested to be due to a plate tectonic slow-down^8^, and lull in plate velocities, during this time. This behaviour is underscored by a dearth of orogenic gold occurrences, VHMS deposits, and iron formations^9^.

The Mesoproterozoic occurs during a period of remarkable climate and surface stability^10,11^. The remarkable consistency of C and O isotopes from c. 1.8 to 0.8 Ga has led to this period being called the "Boring Billion"^10^ or "the dullest time in Earth's history"^11^. Atmospheric O_2_ levels regressed^12^, and seawater sulfate concentrations remained stable^13^. ^87^Sr/^86^Sr gradually declined during this interval, suggesting the contribution of continental erosion to seawater decreased^14^, decreasing phosphorus in the oceans and limiting primary productivity^10^.

**Figure 1:**
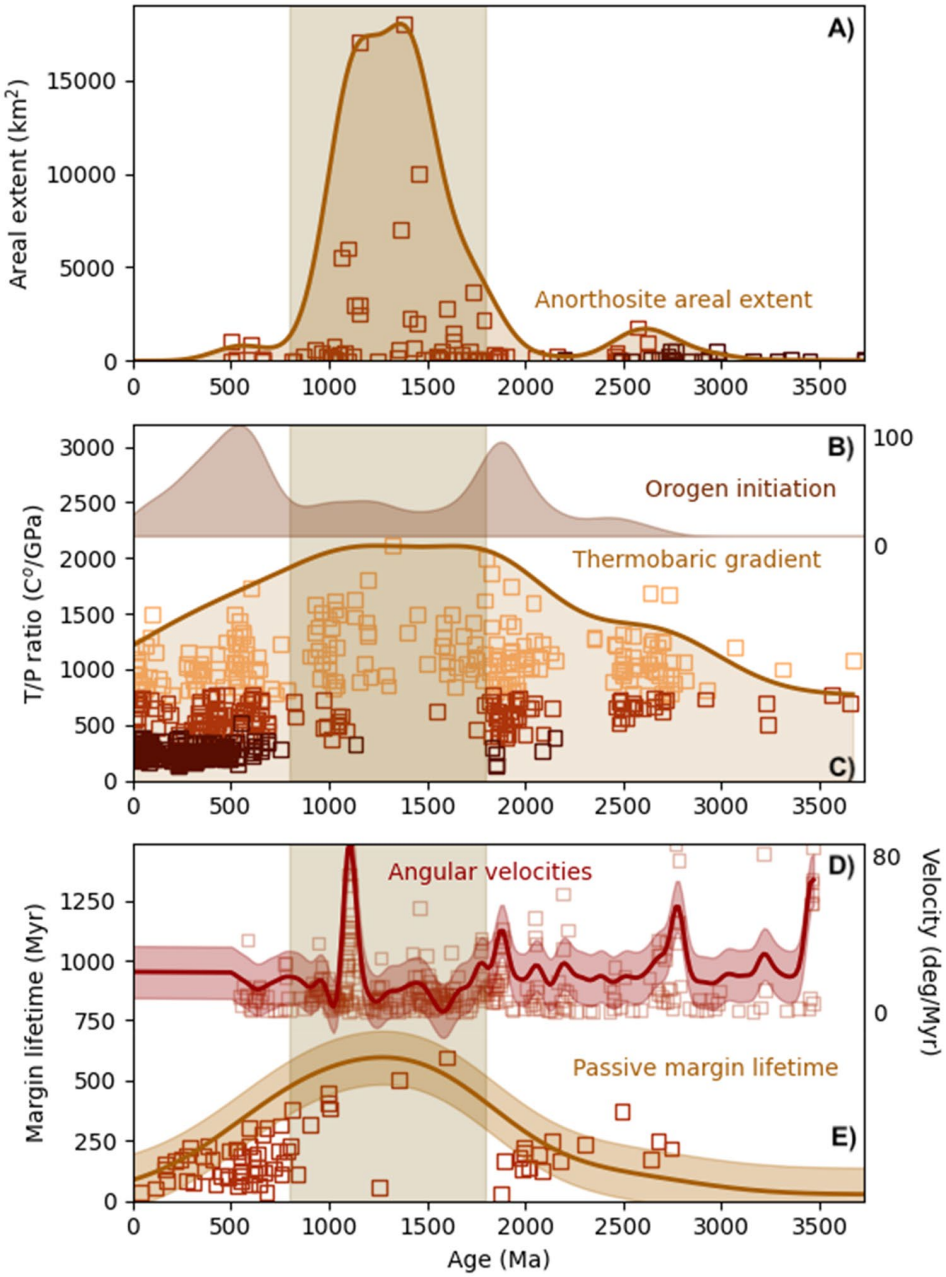
Compilation of constraints on intraplate magmatism, metamorphism and plate dynamics back to the beginning of the Archaean. The period of the "boring billion" from 1.8 to 0.8 Ga is shaded. (**A**) Distribution of anorthosites through time^15^, plotted by areal extent, and a weighted kernel distribution function (kernel width 200 Myr). (**B**) Top shows the weighted kernel density estimate of orogen initiation through time, from the data of Condie^20^ (top, *kw* = 167 Myr). Bottom shows thermobaric ratios (*T/P*) of metamorphism through time, from Brown and Johnson^19^. Shown are data from high T/P localities (including UHT metamorphism (T > 900 °C)) in orange, intermediate T/P localities (red), and low T/P localities (maroon, including UHP metamorphism). Shaded curve shows a weighted KDE (*kw* = 500 Myr) through the high T/P data. (**C**) Top shows angular velocities of APW paths, compiled from a Monte-Carlo sampling of compiled pole paths for the Slave, Superior (plus east Superior), Baltica, South Australian, Kaapvaal, Pilbara, Wyoming and North China blocks (see Supplementary for statistical details). Red line and shaded region show weighted KDE of the data (*kw* = 100 Myr). Bottom shows the lifetime of passive margins from Bradley^7^, and weighted KDE (*kw* = 600 Myr).

Yet whilst the surface and sedimentary cycle stagnated, the magmatic and metamorphic history of the period is remarkable^9^. The Mesoproterozoic is a unique period during which most massif-type anorthosites were emplaced^15^ (Fig. 1A), coincident with anorogenic Rapakivi granites which were largely emplaced during a 500 Myr period spanning the late Paleoproterozoic and early Mesoproterozoic^16^. Both of these rock types are rare in the Phanerozoic or Archaean records. The anorthosites require differentiation from a volumetrically significant basic precursor magma^17^, but evidence of these precursors is rarely found, leading to the suggestion that the magmas were emplaced en-masse in the lower crust or at the Moho^15^. Progressive fractionation of these deep mafic precursors results in feldspar-rich crystal mushes, which may then ascend diapirically and be emplaced in the mid-upper crust^18^. The geodynamic setting of anorthosite production is cryptic. Most anorthosite massifs are argued to have formed in a convergent margin setting^15^, yet it is unclear why they are scarce in Phanerozoic convergent settings or why extensive magmatism in the Archaean failed to produce massif-type anorthosites, suggesting a consilience of factors in the Mesoproterozoic. Furthermore, high T/P (temperature/pressure or thermobaric ratio) crustal metamorphism is dominant in the Mesoproterozoic (Fig. 1B), peaking at over 1500 °C/GPa in the interval 1.5–1.2 Ga^19^.

These magmatic and metamorphic features have been related to secular change in mantle potential temperature^19^, which means they are also coupled to surface tectonics, and are sensitive to global plate velocities (here we generally refer to globally averaged plate velocity). A global plate slow-down^8^ has a number of implications. These include decreased surface tectonic activity, as suggested by the distribution of orogen initiation (Fig. 1B) and passive margin lifetimes (Fig. 1C), environmental stability^10,11^, and inefficient mantle cooling, leading to a warmer mantle, higher-temperature mantle melts and concomitantly higher crustal thermal gradients as recorded by the peak of high T/P metamorphism (Fig. 1B).

Here we test the hypothesis that there was a plate tectonic slow-down using mantle evolution models tightly constrained by the geological record. We benchmark two approaches to deriving global plate motions from the geological record. Paleomagnetic apparent-polar wander (APW) velocities may be used to constrain plate speeds^20^, but they are insensitive to longitudinal motion, and may produce lows due to sparse data sampling^2^. The second, complementary approach is to use the passive margin record, since passive margin lifetimes provide an integrated record of ‘averaged’ plate speeds, and we assess both approaches in the following sections.

## Results

**Figure 2:**
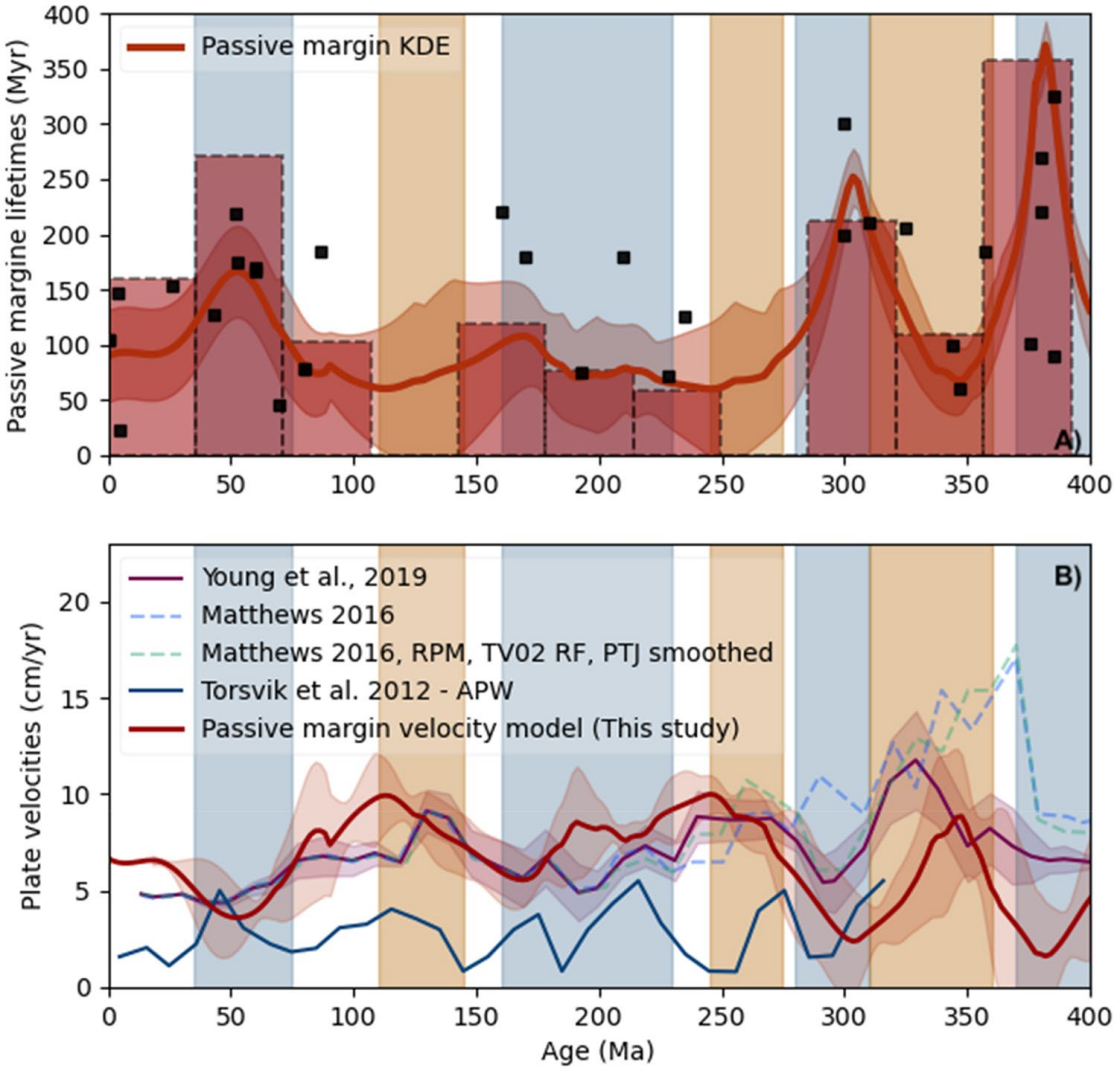
Passive margins and plate velocities in the Phanerozoic. (**A**) Passive margin evolution since 400 Ma (from Bradley^7^) (excluding passive margins that are developing at the present-day). Black squares indicate end of margin lifetime. Red bars show a weighted histogram through the data, with weights calculated from margin lifespans. Red line indicates a weighted kernel density estimate through the dataset, with standard deviations calculated from local data density shaded. Orange and blue divisions indicate periods of short- or long-lived passive margins, respectively. (**B**) Phanerozoic global plate velocity models of Young et al.^48^ (purple), and Matthews et al.^1^ (green and blue dashed line). The orange and blue divisions here correlate with periods of high and low plate velocity, respectively, and anticorrelate with the passive margin ages observed in a). We have used the KDE estimate shown in a to invert for velocity, with the result shown as a thick red line in b. We are able replicate the timing and magnitude of velocity excursions of Young et al.^48^ within the uncertainties of each dataset. Competing velocity models diverge beyond 300 Ma, increasing the uncertainty of the plate estimates. Also, for times less than 50 Ma, passive margin lifetimes are less well constrained, because many are still forming. We have also plotted the APW model of Torsvik et al.^21^ (blue), for comparison with global velocity excursions.

To begin, we constrain the relationship between passive margin lifetimes, and plate tectonic velocities, which are shown in Fig. 2. Periods of long passive-margin lifetimes are clearly correlated with global velocity downturns (highlighted by the shaded blue regions). Conversely, periods of short passive margin lifetimes correlate with periods of fast plate velocities (orange regions). This is expected as plate ages at subduction, or passive margin lifetimes, are inversely related with plate speeds. In contrast, global apparent polar wander paths^21^ (APW, blue curve in Fig. 2B) systematically underestimate plate velocities, and show a fairly poor correlation with trends in global plate circuit velocity. Slow velocities are expected, as APW paths can be insensitive to longitudinal variations in plate motion. The lack of correlation between the two, however, may be due to APW coming largely from continental regions, which both underestimates plate velocities compared to oceans, and constitutes only a small fraction of Earth’s total surface area^22^.

For comparison, we have also used the passive margin timeseries to construct an inverse fit, and the resulting velocity model, based on passive margin lifetimes, is shown in red in Fig. 2B. Within the uncertainty of these datasets, the modelled velocities fit the observed velocities well. For our modelling, the average plate age at a subduction zone is the most relevant parameter, and correspondingly passive margin ages at the date of their cessation are considered here. However, for long-lived passive margins this introduces some uncertainty in the timing. Commonly, passive margins with ages less than 50 Myr are still developing, so these are excluded from this analysis; consequently, the fit for < 50 Myr is poorly-constrained. For other periods, the velocity model follows the trend in observed global plate velocities, with some divergence for older ages where global plate velocities are more difficult to determine.

**Figure 3:**
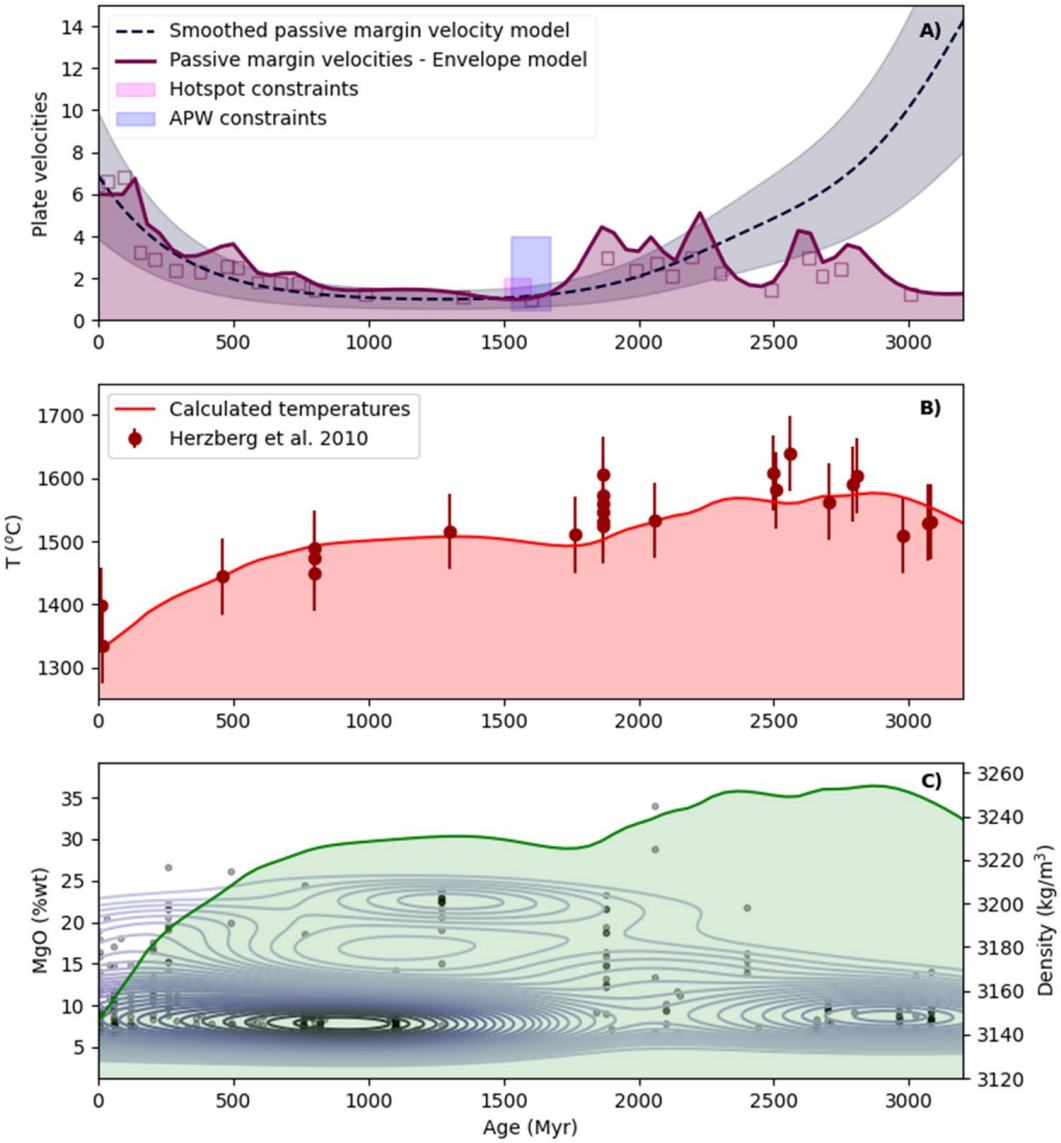
(**A**) Estimated velocities based on passive margin longevity^11^. Blue dashed line shows smoothed KDE fit (kw = 600 Myr), shaded region ± fractional STD. Purple line shows velocities based on an envelope model (ie. calculated maximum lifetimes in a given interval). Blue box shows (minimum) velocity constraint from the APW data (see Supplementary), and magenta box shows velocity constraint from a Proterozoic hotspot track. (**B**) Calculated mantle temperatures using plate velocities derived from (**A**), using a parameterised convection approach. Red circles indicate mantle temperature estimates from Herzberg et al.^23^ with notional ± 60 °C uncertainty. (**C**) Gabbro distribution (plotted against MgO, left) shown as a KDE, with individual data points, sourced from the GEOROC database. Green curve indicates melt densities calculated from the mantle temperature evolution shown in (**B**), assuming compositions were melts.

Based on the correlation between passive margin lifetimes and velocities in the Phanerozoic, we use the relationship to estimate average plate velocities throughout the Proterozoic, for which there are few plate-speed estimates. Our results are shown in Fig. 3A. Two fitted curves are shown, one for the smooth inversion of the KDE of passive margin ages shown in Fig. 1C, and another for an ‘envelope’ model (effectively a Hilbert transform of the regularised data). The KDE in this case is adversely affected by the sparse data density in the Archaean, pulling the estimated Archaean velocities to overly high values. The envelope model, designed to prevent this effect, represents the data better, with the caveat it is sensitive to data noise. The average plate velocities for the Mesoproterozoic estimated in this way are of the order ~ 1–1.5 cm/yr (compared with ~ 5.5 cm/yr today). This estimate compares well (Fig. 3A) with the only direct constraint on Proterozoic plate velocities of 1–1.5 cm/yr, determined using an Australian Proterozoic hotspot track^6^, and falls within the range for minimum velocities determined from APW data (see Supplementary).

For the calculated surface velocities, we can calculate mantle temperature back to the Paleoproterozoic, assuming standard mobile-lid convective scaling^22^, and integrating temperature back from the present day. The detailed equation set is outlined in the methods, and the code is provided in the supplementary. The key addition here is that plate velocities are set from geological constraints, which impacts the rate of heat loss from the system. Surface heat loss, together with radiogenic heat production, determines the system temperature rise or fall in a given timestep, and thus the temperature evolution of the interior. This calculation is valid for periods during which plates were mobile (though not for complete shutdowns). The results are shown in Fig. 3B (for the envelope model in 3a), together with petrological estimates of mantle potential temperature (*T*_p_)^23^. For the Proterozoic, the modelling predicts the petrological estimates of mantle *T*_p_ well. The strong decrease in mantle temperature over the last 1200 Myrs is due to efficient plate tectonic cooling. Prior to that, the slow increase in mantle temperature from 1800 Myr is largely due to slower plate velocities, as predicted by the long passive margin lifetimes, following a period of decreasing mantle temperature since 2400 Myrs. However, we note that the temperature scaling we use will not apply to alternate tectonic regimes, which may have existed on the early Earth^24^. For the Proterozoic–Phanerozoic after 2.3–2.2 Gyr, when plate tectonics is thought to have been operating^25^, the variations in mantle *T*_p_ are largely related to fluctuations in the surface velocities of plates and associated convective cooling.

The variation in mantle *T*_p_ from the present day back to the early Paleoproterozoic has implications for the density of mantle-derived melts. Higher mantle *T*_p_ generates melts with higher Mg#^26^, which have a higher density. Therefore, these melts may reach their level of neutral buoyancy, and be emplaced more commonly at the Moho or in the lower crust than melts derived from mantle with lower *T*_p_. In the crust, the depth at which melts are emplaced is a function of the relationship between melt density and the density structure of the crust, both of which have compositions and, therefore, densities that have evolved through time^27^. Direct estimates of the composition and density of these melts as they were emplaced in the crust is not possible. As a proxy we use gabbro occurrences through time, which show both high densities and a high frequency during the Proterozoic—although many are cumulates and are not strictly melt compositions.

Variations in the ratio of extrusive to intrusive magmatism is frequently cast as the intrusive:extrusive ratio^28^, or, here, as the proportion of extrusive to total magmatic volume (E/T). Crisp^28^ and White et al.^29^ documented this for a number of magmatic and tectonic environments, and proposed that an E/T ratio of 0.21 ± 0.10 is common to many magmatic systems given the large uncertainties involved in such estimates. For our analysis, we focus on intracontinental volcanism as it is generally well preserved in both contemporary and Proterozoic settings (in contrast with oceanic volcanism, which is not preserved), and plate-boundary volcanism may be influenced by tectonic rates, e.g., the rate of rifting, and thus it is not an independent constraint on mantle temperature. The most tightly constrained intracontinental intrusive system of White et al.^29^ (Yellowstone) has an E/T ratio 0.25. Notwithstanding the large uncertainty, this ratio is expected to be relatively higher at the present day and lower for hotter mantle conditions, as a higher proportion of mantle-derived melts are emplaced in the deeper crust. A higher proportion of intrusive magmatism would have the predicted effect of elevating crustal thermal gradients, as well as providing the large volume of precursor basic magmas necessary to drive the production of anorthosites by fractional crystallisation.

**Figure 4:**
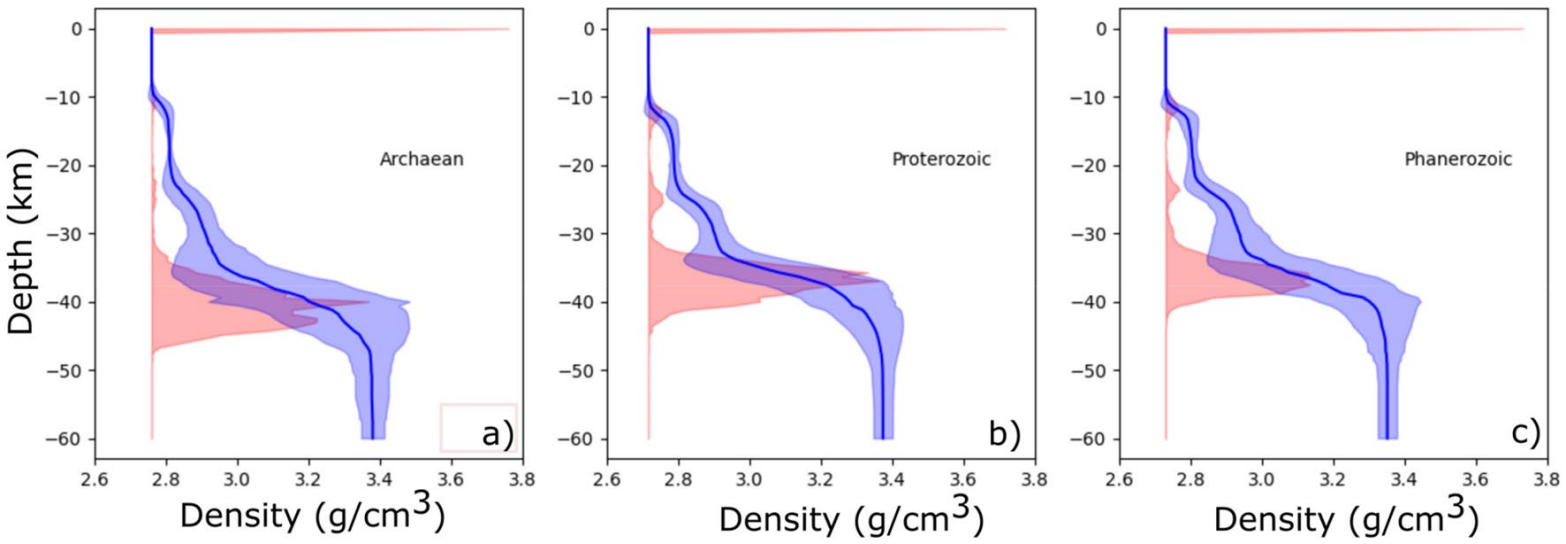
Crustal density models (blue, ± 1SD) for averaged (**a**) Archaean, (**b**) Proterozoic and (**c**) Phanerozoic crust, from the CRUST1.0 seismic model. Red indicates the distribution of melt, calculated using a Monte Carlo approach from the melt densities shown in Fig. 3C.

Melts ascending from the mantle are driven primarily by their positive buoyancy rather than overpressure^30^, and we consider this a fundamental requirement for melt ascent. Variations in mantle-derived melt density arise due to local differences in temperature, composition and volatile content, as well as fractional crystallisation on ascent and crustal assimilation. Many of these processes drive melts to lower densities, and these variations may be modelled using a skew distribution of melt density (see Supplementary for details). Crustal densities are calculated from the CRUST1.0 seismic model^31^, using global averages for representative crust of Phanerozoic, Proterozoic and Archaean age. These derived crustal density profiles, together with uncertainties of ± 1 S.D., are shown as blue envelopes in Fig. 4. The emplacement depths of mantle-derived melts are then calculated using a Monte-Carlo approach, where a melt-density distribution based on the mean mantle temperature is used (Fig. S3). Discrete batches of melt drawn from this distribution are allowed to propagate up through the mantle and crust until each reaches a level of neutral buoyancy, where it is emplaced. In each calculation the crustal density profile is randomised, drawing values from the uncertainty envelope at each depth. The calculation is repeated thousands of times (see Supplementary), and in this way a statistical distribution of emplacement depths is achieved, encapsulating the main variations in melt and crustal densities, and emplacement depth. The distribution of melt emplacement is shown as red envelopes in Fig. 4. The hotter temperatures of the Archaean and Proterozoic result in more intrusive melt than is emplaced in the Phanerozoic. However, in the Archaean this intrusive melt is largely emplaced at the base of the crust, whereas in the Proterozoic a greater proportion is emplaced at the mid and upper crust interfaces—a function of the sharper density interfaces of average Proterozoic compared to Archaean crust (blue lines).

**Figure 5:**
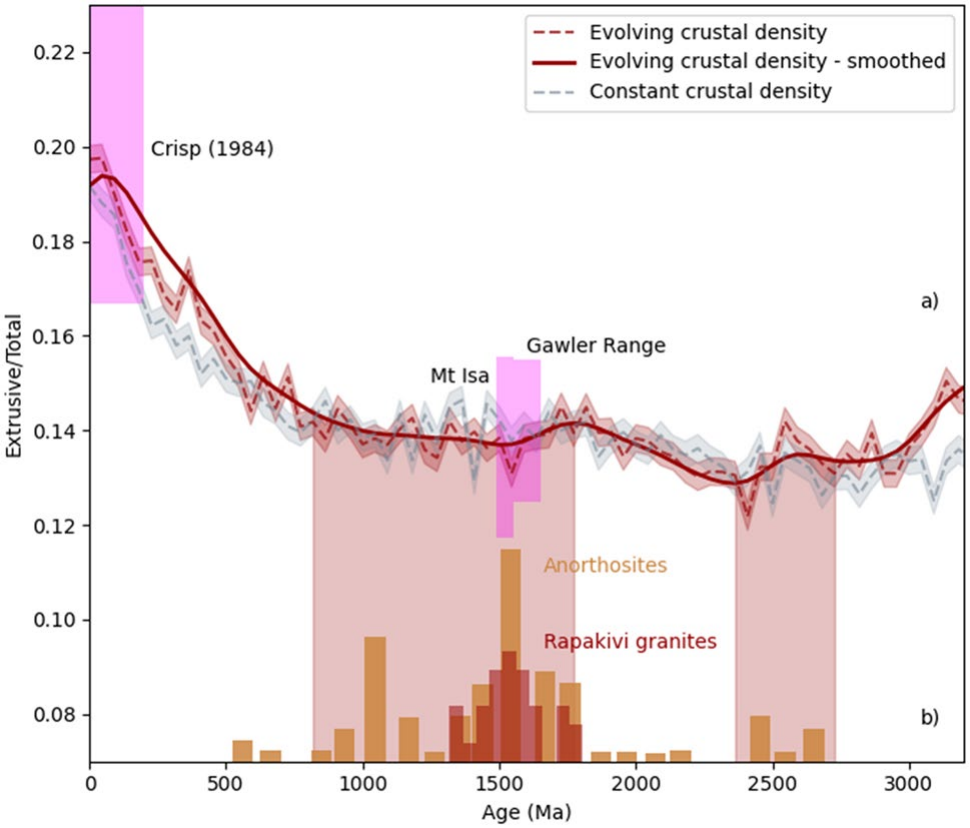
(**a**) Evolution of extrusive to total magmatism through time (E/T ratio), calculated for an evolving crustal profile (red) and for the case where the crustal density profile does not change (a constant Proterozoic profile) and only the mantle-derived melt densities change with time (teal). The latter isolates the effects of mantle-temperatures alone. Uncertainties are shown by shading, and a polynomial fit by the dark line, indicating two minima at ca 800–1800 Ma, and ca. 2300–2700 Ma. (**b**) Distribution of anorthosites^15^ and Rapakivi granites^49^ peaking in the Mesoproterozoic, similar to a low in the E/T ratio. A minimum in the E/T ratio occurs across the Proterozoic–Archaean boundary corresponding to the limited number of late-Archaean/early Proterozoic massif-type anorthosites. Magenta regions show the E/T ratio for intraplate volcanic systems from White et al.^29^ on the left, and those calculated here for the Gawler Range Volcanics and Mt Isa based on geophysical modelling.

The relative evolution of the ratio of extrusive melt to total magmatic products (E/T) through time is shown in Fig. 5. For the Phanerozoic example (Fig. 4c), the blue envelope for density shows a clear difference between upper, middle and lower crust, before transitioning to the mantle. The melt emplacement envelope demonstrates significant intrusion at the mantle–crust density interface, smaller amounts of melt emplaced at the lower–middle and middle–upper crustal density interfaces, and then a spike of melt erupting at the surface (Fig. 4c). The E/T in this case is around 0.2 (Fig. 5). For the Proterozoic example, significantly more melt is emplaced at the crust–mantle boundary as a function of the higher density of melt derived from a hotter mantle (Fig. 4b), and the E/T ratio is around 0.15 (Fig. 5). In Fig. 5, the blue curve incorporates both the evolving mean melt density (Fig. 3C) and the crustal density profiles (Fig. 4). The red curve isolates the effect of melt density, by holding the crustal profile constant to that of the Proterozoic. The substantive effect in both cases is an E/T minimum due to higher mantle temperatures in the interval c. 1800–1000 Ma, and also at ca. 2500 Ma—another period of unusual massif anorthosite production.

As confirmation that the relative low in the Proterozoic is realistic, we have revisited two Proterozoic volcanic systems in Australia, the Gawler Range volcanics and Mt Isa Inlier in the Eastern Fold Belt, for which an estimate of E/T can be made. Both systems represent intracontinental volcanism, with dominantly felsic shallow-crustal/eruptive sequences, although the volcanic sequence in Mt Isa is complicated by the contemporaneous tectonics of the ca. 1500 Ma Isan orogeny. Recent deep exploration seismics in the Mt Isa region, Queensland^32^, have been digitised allowing the volume of deep intrusive units to be inferred, which complements the volume estimates of volcanic rocks made from mapping. In the case of the Gawler Range volcanics (GRV), South Australia, the surface flows have been mapped extensively, and volume estimates made on the flows. The GRV is coincident with a gravity high, previously interpreted to be an associated mafic intrusion in the middle crust. We have re-modelled the gravity anomaly (see Supplementary), to estimate the size of the intruded mafic body, and then use this to calculate the E/T ratio. In both cases, there are uncertainties on the original extent and degree of preservation of the volcanics, and on the full identification and estimate of size of the subsurface intrusives; these uncertainties are reflected in the scale of the magenta bars in Fig. 5. These two magmatic systems exhibit low E/T ratios consistent with the lower estimates modelled for the Proterozoic.

## Discussion

Variations in E/T through time explain one of the paradoxes about the Mesoproterozoic. Whilst the crust is widely known to have been hot^19^, there is little excess volcanism to account for this^9^. Certainly, hotter mantle temperatures are expected to generate more voluminous magmatism, yet this is not seen in the surface volcanic record^33^. This paradox is resolved by consideration of the secular variation in E/T—large amounts of mantle-derived melt certainly were generated, as required for anorthosite genesis and demonstrated by the anorogenic Rapakivi granites and associated basalts. However, the density of the mantle-derived parental melts probably ensured they were largely emplaced in the deeper crust or at the Moho. This advected heat contributed to the high implied thermal gradients in the Mesoproterozoic^19^, and fractionation of some of these melts was responsible for the anorthosite bloom which is characteristic of this period. In addition, emplacement of the Rapakivi granites correlates with the emplacement of anorthosites (Fig. 5), peaking in the Mesoproterozoic. Similar to the anorthosites, formation of the rapakivi granites has also been associated with extensive underplating of basic melts^15,16^ and subsequent magma mingling^34^.

The style of magma emplacement and the evolution of the E/T ratio can have a profound effect on the cooling of the Earth^35,36^. At the extreme, the models of Moore and Webb^35^ demonstrate a 'heat-pipe' regime—a high E/T endmember—where most of Earth's internal heat is removed by effective extrusive volcanism, moderating extreme Archaean mantle temperatures. More moderate regimes with lower E/T ratios have shown a style of "squishy-lid" tectonics^36^, a high temperature distributed deformation style that may have some commonality with the observed Mesoproterozoic peak for high T/P metamorphism. The transition between these regimes may have been moderated by an evolution in crustal composition in the Proterozoic^27^, changing crustal thickness^37^, and more defined upper and middle crustal density boundaries. The ratio of upper-mid crust/lower crust intrusions is ~ 4 times more in the in the Proterozoic, compared to the Archaean, where the preponderance of intrusive magmatism is at the crust–mantle boundary (Figs. 4, S4). This change to a higher proportion of intracrustal emplacement of intrusive magma in the Proterozoic may explain the scarcity of massif-type anorthosites in the Archaean.

The Mesoproterozoic is associated with world-class ore deposits, including Mt Isa^38^ in Queensland, and Olympic Dam^39^ in South Australia, which both seem to be associated with modified lithospheric signatures, and lower crustal emplacement of basic magmas. These lithospheric-scale ore systems seem to be powered at least in part by the heat provided by the basic intrusions, as well as by the specific composition of the mobile fractionated products, including interaction of the crust with released fluids—leading, for instance, to the enormous enrichment in radioactive species such as uranium at Olympic Dam^39^. This enrichment in radioactive elements is a peculiar feature of Proterozoic Australia^40^, and is associated with extreme radiogenic heating, high crustal heat flow and tectonic weakening. In conjunction with underplating of basic melts and advective heating of the crust, this extreme radiogenic heating has contributed to the dominance of high T/P metamorphism during this period in Australia.

The Proterozoic has been associated with early development of a supercontinent cycle, with assembly of the supercontinent Nuna/Columbia initiating around 1.9 Ga, and final assembly around 1.7–1.5 Ga, followed by the assembly of Rodinia from 1.2 Ga^20^. It has been suggested that supercontinents could blanket the mantle beneath them^41,42^, resulting in locally elevated mantle temperatures, and enhanced crustal volcanism—potentially resulting in similar predictions to a tectonic lull^43^. Differentiating between the relative importance of these two mechanisms requires more detailed tectonic reconstructions of Proterozoic supercontinent configurations and duration^20^. Further evidence for the association of mid-Proterozoic high thermobaric gradients, ore genesis, and increased intrusive emplacement, could be garnered from detailed analysis of magmatic and metamorphic systems globally, including detailed metamorphic thermodynamic modelling^47^, regional volcanic volume estimates, and detailed mid-crustal geophysics to constrain emplacement systems. In addition, more detailed examples of E/T volcanism more generally throughout the Proterozoic are needed to better constrain this important diagnostic of Earth’s magmatic and thermal evolution.

A lower middle-Proterozoic E/T ratio would imply not only a lower rate of volcanic activity, but also a lower rate of volcanic degassing. Volcanism is one of the major sources of CO_2_ and other greenhouse gases in the atmosphere. However, during the Mesoproterozoic, slower plate motions would have led to diminished volcanic output, lower rates of orogeny, and likely subdued volcanic arc topography, and possibly mountain height^44^. Weathering of extreme topography is a major CO_2_ sink via the CO_2_-silicate weathering cycle^45^. Together, these factors suggest an unusual feedback between geologically subdued CO_2_ sources and sinks in slow plate modes, as lower CO_2_ degassing is complemented by commensurate lower CO_2_ drawdown. The diminished tempo of CO_2_ sources/sinks in a plate slowdown predicts subdued climatic fluctuations, and periods of extreme climate stability, which are observed during the Mesoproterozoic. Furthermore, this feedback predicts a decreased nutrient influx into the oceans during this time, which is congruent with the strontium isotopic record^14^, decreasing oxygen^12^, and limited primary productivity throughout the Mesoproterozoic^10^. The breakup of the supercontinent Rodinia after ca. 800 Ma^46^, associated with the development of modern, cold subduction systems^47^ may have finally provided the erosional mechanism to end the "boring billion", and initiate a snowball Earth via associated CO_2_ drawdown.

## Methods

### Proterozoic intrusive magmatism

The calculation of Proterozoic extrusive:instrusive ratios used either deep seismics^32^ or the National Australia gravity grid to determine the extent of intruded bodies. Surface volcanics were determined from prior geological mapping, and seismic constraints. From the Mt Isa deep seismic transects we calculated the cross-sectional area of each volcanic/magmatic geological unit, based on a digitised model (see Supplementary information), and calculated the ratio of extrusive volcanics to deep subsurface magmatic bodies. For the Gawler Range volcanics, we used the National spherical cap Bouguer gravity maps (source listed in Supplementary) to construct 2D and 3D transects, and utilised a forward-tesseroid model in a Monte-Carlo framework to determine the likeliest size of the associated anomaly, and associated standard deviations. The full details and the link to the code used are included in the Supplementary.

### APW velocities

Paleomagnetic APW velocity paths were constructed using the Paleomagia database. We filtered out poles for each craton using python and Pandas, retaining poles with a cumulative score of 3 or higher^50^, with defined A95, and with age uncertainties less than 150 Myr. Poles were also filtered for minimum angular distance between to account for reversals. We utilised the Pmag.py library to construct a statistical Fisher distribution of poles, and a truncated normal distribution of their ages. Then we used a Monte Carlo approach to calculate angular distance between these pole pairs, and the average age, giving a distribution of angular velocities, which we compile and then run through a weighted Gaussian kernel filter with a halfwidth of 150 Myr to construct an apparent polar wander velocity curve, with standard deviations. Full details of the method and the code used are provided in the supplementary.

### Crustal model and melt emplacement

We utilised the database CRUST1.0^31^ to extract local crust of Archaean, Proterozoic, and Phanerozoic ages. Examples of different terrane average density profiles are plotted in the supplementary. We aggregate crust types using a weighted scheme for each time period (see code for details), to construct average crustal structures, for surviving crust, for Archaean, Proterozoic, and Phanerozoic terranes. The statistical crustal profile distributions, plus standard deviations, are then sampled to calculate random crustal profiles used in the Monte-Carlo melt propagation calculations.

The depth of melt emplacement is assumed to correlate with the level of neutral buoyancy for mantle-derived melts, which is a function of the crustal density profile, and the melt density. The mean melt density is calculated for the potential mantle temperature at different times, using the relationship of van Thienen et al.^26^:

1 $${\rho} = { 15}00 \, + { 1}.{925}\left( {{\text{T }} - { 273}} \right) \, + { 5}.{153 } \times {1}0^{{ - {4}}} \left( {{\text{T}} - {273}} \right)^{{2}}$$

We assume this density defines a skewed distribution (see Supplementary); we randomly draw melt packets from this distribution, and calculate the level of neutral buoyancy for this melt in a randomly sampled crustal density profile.

### Thermal evolution model

We use a parameterised thermal evolution model, based on a set of conservative equations, to calculate mantle temperatures back through time. The global energy conservation equation is:

2 $${C}_{Earth}\frac{dT}{dt}=H\left(t\right)-Q\left(t\right)$$

Here C_Earth_ is the heat capacity of the Earth (~ 7 × 10^27^ J/K), T is the mantle potential temperature, H the average heat production of the Earth, and Q is surface heat loss. Heat production through time is calculated from

3 $$H={\sum }_{i}{C}_{0}^{i}{H}^{i}exp\left(\frac{tln2}{{\tau }_{1/2}^{i}}\right)$$

Here i refers to the radioactive isotope (^238^U, ^235^U, ^40^K or ^232^Th), C the initial isotope concentration, t is time, and τ_1/2_ the appropriate half-life. Note that H in Equation M3 is scaled to total Watts for the mantle's mass (4 × 10^24^ kg).

We integrate Equation M2 backwards in time. In contrast to traditional parameterised models, though, we impose the lifetimes of passive margins as a constrain on plate age, and thus velocity.

We utilise the relationship:

4 $$v=\frac{\partial d}{\partial t}{Ra}^\frac{2}{3}$$

We assume the distance travelled ($\partial d$) does not systematically vary in time. We also assume that passive margin lifetimes (t_PM_) act as a proxy for the time a plate takes to reach a subduction zone ($\partial t$). This allows to exploit the scaling relationship between Nusselt number (Nu) and Rayleigh number (Ra):

5 $$Nu\approx {Ra}^\frac{1}{3}\Rightarrow {Nu}^{2}\approx {Ra}^\frac{2}{3}\frac{1}{{t}_{PM}}\Rightarrow Nu\approx \frac{1}{{{t}_{PM}}^{1/2}}$$

The proportionality between Nu and 1/${{t}_{PM}}^{1/2}$ is determined by fitting the present-day heat flux Q (including a lag). Nusselt number can be converted into global heat flux Q by multiplying by the conductive heat, given by kAΔT /d, where k is thermal conductivity (3.5 W/m K), and A the surface area of the globe (given by 4πr^2^). Once Q is determined for this timestep, and H known, the change in temperature can be determined (Equation M2). This temperature change is added back into the mantle, warming it up as we advect backwards. Full details and code are linked in the supplementary, and data and scripts are available in the accompanying repository 10.5281/zenodo.6179433.

## Supplementary Information

Supplementary Information.

## Data Availability

Full details and code are linked in the supplementary, and data and scripts are available in the accompanying repository 10.5281/zenodo.6179433.
