# Supplementary material for: Earth's anomalous middle-age magmatism driven by plate slowdown
Source: Sci Rep. 2022 Jun 21;12:10460. doi: 10.1038/s41598-022-13885-9 (PMC9213423; doi:10.1038/s41598-022-13885-9)
Supplement: Supplementary file 1 — Supplementary Information. [file 41598_2022_13885_MOESM1_ESM.docx]

**Supplementary Information**

**S1 Kernel density estimates of datasets**

We use a weighted Gaussian kernel density estimate to highlight trends within the datasets shown in Figures 1 and 2. The kernel takes the form

$$W=\frac{Z}{\sqrt{\pi r}}e^{-\left( \frac{r}{h} \right)^{2}}$$

Here Z is the value of the input data, h the kernel halfwidth, and r the radius from the data considered by the Kernel. The result is summed over the data within h distance of the calculation point. Note: i) r is typically the absolute value of the difference in time between data points in the cases herein, ii) a Gaussian kernel typically extend a distance of 3h from the calculation point, and this total distance must be considered to avoid truncation errors. In the cases herein h is varied to account for different data densities, and these cases are explicitly noted in Figure 1. The approach is similar to adaptive kernel lengths in smooth-particle hydrodynamic simulations, where if the default kernel width is not sufficient to ensure a minimal data density (eg. 3 surrounding data points in the plots herein), then the kernel width is incrementally increased until this condition is met. In some cases, the data has a mean removal to facilitate visualisation. Some examples of the implementation are found in the repository (eg. S2_ii_plot_APW_vels.py).

**S2 Statistical details of APW velocity calculations**

We used the Paleomagia database to filter out poles for each craton using Pandas with python, and retain poles with a cumulative score of 3 or higher, with defined A95, and with age uncertainties less than 150 Myr. The selected poles are available in the file paleopoles.zip in the zenobo repository. We have also heavily used the Pmag.py library ([https://pmagpy.github.io](https://pmagpy.github.io/)) in the calculations outlined below.

We statistically sample a Fisher distribution of poles.

i) Send the precision values, K, to fshdev to calculate declination and inclination (loop N times to obtain a distribution of points for each pole).

ii) Rotate these declination/inclination tuples to the reference frame defined by the pole's longitude and latitude (using dodirot).

iii) For each pole age, use the upper and lower bounds to define a truncated normal distribution, and sample N ages from this distribution.

iv) Save the (dec, inc, age) sampled distributions as arrays for each pole.

v) For each successive pole (ie. P[i] and P[i+1]), and for each point, N, in the distribution, we average the age, calculate the angle between each (dec, inc) pair (using pmag.angle), calculate the time interval, and then average angular velocity by dividing the angle by the time interval. The result is N calculations of angular velocity for each pole pair. The mean and standard deviation are calculated from this, for each time (ie. (age[i]+age[i+1]) / 2.0).

vi) Concatenate the APW arrays for each craton into a global path, and calculate either a LOWESS fit or weighted kernel estimate to the data. The result is shown below.


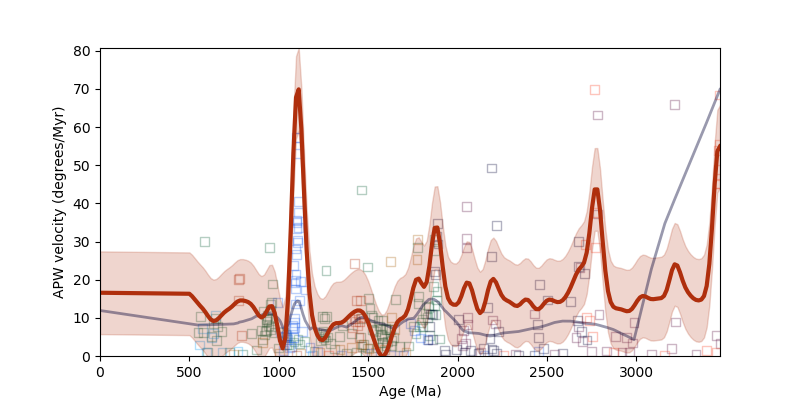


Figure S1. Plot of Apparent Polar Wander angular velocities vs time, from the procedure outlined above. Red line represents a weighted kernel density estimate of the data, with a Gaussian kernel halfwidth of 150Myr. The blue line represents a LOWESS fit from the Python library statsmodels (note it has difficulty at boundaries). APW velocities were only calculated for the Precambrian, Phanerozoic values are held constant.

**S3 Thermal evolution models**

The starting point for most parameterised thermal evolution models is a set of conservative equations. We begin with the global energy conservation equation:

$$C_{Earth}\frac{dT}{dt}=H\left( t \right)-Q\left( t \right)$$

(Equation S1)

Here C_Earth_ is the heat capacity of the Earth (~7x10^27^ J/K; Korenaga, 2003), T is the mantle potential temperature (see Christensen (1984) and Korenaga (2003)), H the average heat production of the Earth, and Q is surface heat loss. Heat production through time is calculated from

$$H=\sum_{i} C_{0}^{i}H^{i}exp\left( \frac{tln2}{\tau_{1/2}^{i}} \right)$$

(Equation S2)

Here i refers to the radioactive isotope (^238^U, ^235^U, ^40^K or ^232^Th), C the initial isotope concentration, t is time, and τ_1/2_  its half-life. Note that H in Equation S2 is scaled to total Watts for the mantle's mass (4x10^24^ kg). These parameters are listed in Table S1.

| **Isotope** | **H (W/kg)** | **τ _1/2_ (yr)** | **C (kg/kg)** |
| --- | --- | --- | --- |
| **^238^U** | 9.46 x 10^-5^ | 4.47 x 10^9^ | 30.8 x 10^-9^ |
| **^235^U** | 5.69 x 10^-4^ | 7.04 x 10^8^ | 0.22 x 10^-9^ |
| **^232^Th** | 2.64 x 10^-5^ | 1.40 x 10^10^ | 124 x 10^-9^ |
| **^40^K** | 2.92 x 10^-5^ | 1.25 x 10^9^ | 36.9 x 10^-9^ |

*Table S1: Heat producing isotopes and their properties (adapted from Turcotte and Schubert (2002)).*

The initial potential mantle temperature is for the present day (1673K average for the whole mantle). We integrate Equation S1 backwards in time. In contrast to traditional parameterised models, though, we do not calculate the viscosity or Ra for a given temperature, to calculate the Nusselt number. Instead, we impose the lifetimes of passive margins as a constrain on plate age, and thus velocity.

We utilise the relationship (Turcotte and Schubert (1982)):

$$v=\frac{\partial d}{\partial t}{Ra}^{\frac{2}{3}}$$

(Equation S3)

We assume the distance travelled ($\partial d$) does not systematically vary in time (plate models show little systematic variation in time - see O'Neill et al. (2020), also c/w Maillard et al (2020)). We also assume that passive margin lifetimes (t_PM_) act as a proxy for the time a plate takes to reach a subduction zone ($\partial t$). This allows us to exploit the scaling relationship between Nusselt number (Nu) and Rayleigh number (Ra):

$$Nu\approx{Ra}^{\frac{1}{3}}\Rightarrow{Nu}^{2}\approx{Ra}^{\frac{2}{3}}\frac{1}{t_{PM}}\Rightarrow Nu\frac{1}{{t_{PM}}^{2}}$$

(Equation S4)

The proportionality between Nu and 1/${t_{PM}}^{2}$ is determined by fitting the present-day heat Q. Nusselt number can be converted into global heat flux Q by multiplying by the conductive heat, given by kAΔT /d, where k is thermal conductivity (3.5 W/m.K), and A the surface area of the globe (given by 4πr^2^). Once Q is determined for this timestep, and H known, the change in temperature can be determined (Equation S1). This temperature change is added back into the mantle, warming it up as we advect backwards. The implementation is provided in the code S3_i_parameterised_evolution.py in the Repository package.

**S4 Crust model and melt transport calculation**

*Crustal models*

We downloaded the crustal dataset Crust1.0 of Laske et al. (2013). The URL for download is: <https://igppweb.ucsd.edu/~gabi/crust1.html>

We used the Crust 1.0 model to extract crustal density profiles of terranes of different ages from around the world. Our script to process this data is found in the Repository package (S4_i_compile_crust.py).

We utilised the following crust types:

#Crust types

# F- 03: Archean (Antarctica)

# G1 04: early Archean

# G2 05: late Archean

# H1 06: early/mid Proter.,

# H2 07: early/mid Proter. (Antarctica, slow)

# I1 08: late Proter.

# I2 09: slow late Proter.

# Z1 24: Phanerozoic

# Z2 25: fast Phanerozoic (E. Australia, S. Africa, N. Siberia)

#D- 01: Platform

We have plotted average crustal density profiles (+/- 1 standard deviation, shaded region) for some of these examples below.


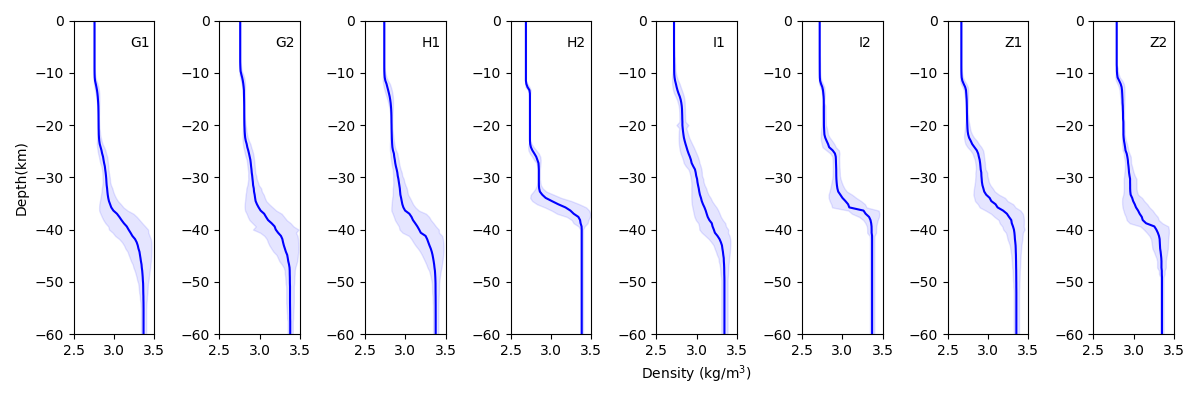


Figure S2 Average crustal density profiles (+/- 1 standard deviation, shaded blue region) for Archean (G), Proterozoic (H, I) and Phanerozoic (Z) crustal terranes, from the Crust 1.0 model.

We compiled weighted averages of these types of crust to represent crustal density for three different Eons (Archaean, Proterozoic, and Phanerozoic). We construct a Monte Carlo simulation where randomised density distributions are constructed on each iteration from the distributions for each crustal terrane described above. At the same time, we calculate the mean melt density for the potential mantle temperature at different times, given by the relationship of van Thienen et al. (2004):

ρ = 1500 + 1.925(T - 273) + 5.153x10^-4^ (T-273)^2^

(Equation S5)

We then assume that the melt density represents a distribution. This comes from the observation that melts may cool as they rise (increasing their density) but also, and more significantly, undergo extreme fractionation or assimilation, decreasing their density, as well as processes like volatile dissolution and bubble nucleation, etc. They may also preferentially rise under favourable stress regimes. Here we largely consider the effects of buoyancy forces. These variations in melt density can be constrained heuristically from observations of volcanic rock density driven (originally) by mantle melting, in arcs, for example. The empirical relationship of the sort described can be represented by a gamma distribution, and a representative melt distribution (for a melt with an original mantle density of 3100 kg/m^3^) is shown in Figure S3.


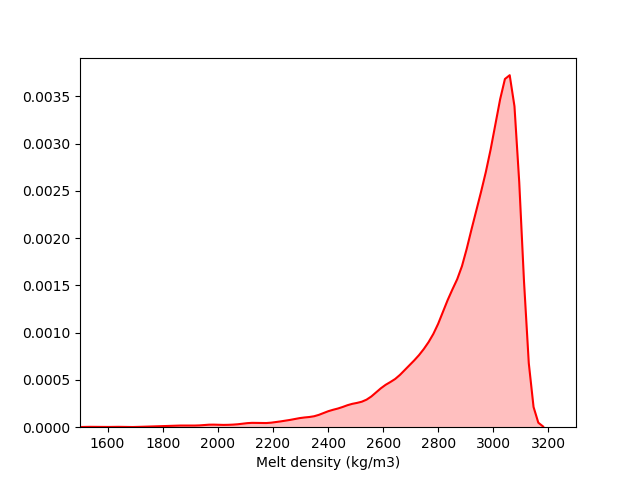


Figure S3. Distribution of melt density, for ascending melt packets in the Monte Carlo simulation of melt transport, for a mantle melt of original density 3100kg/m^3^. Fractionation and other processes drive melts to lower density, and these deterministically complex processes are captured here in a statistical skewed (gamma) distribution.

The results of this are shown in Figure 4 in the main text, and our code for the simulation is in S4_ii_IntExt_Calc.py in the Repository package.

Lastly, we consider the variation in upper- and middle-crustal intrusions, compared to intrusions in the lower crust or at the crust–mantle boundary. The results are shown in Figure S4.


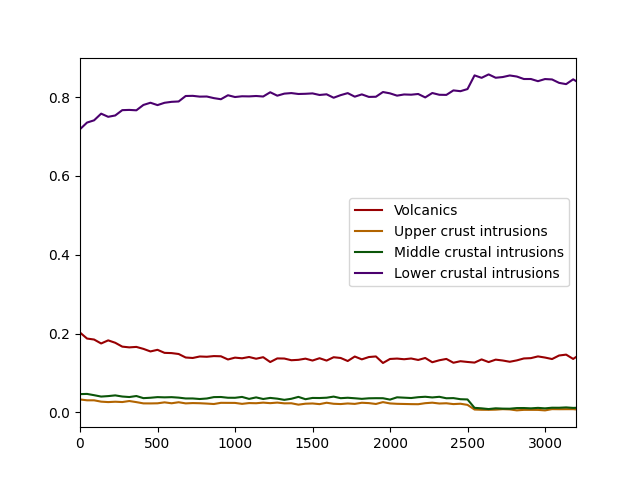


Figure S4. Relative proportions of extrusive volcanics, and upper, middle, and lower crust/crust–mantle boundary intrusions through time, for the same data shown in Figures 4 and 5. The change in crustal density from a typical Archaean profile, to a mature Proterozoic profile, has an apparent affect, promoting more upper- and middle-crustal magmatic emplacement, particularly of fractionated melts such as anorthosites.

If we consider the ratio of upper- and mid-crustal intrusions, to lower-crustal intrusions, this number is 0.1101 for the Phanerozoic, 0.07315 for the Proterozoic, and 0.01793 for the Archaean. The change in crustal structure in the Proterozoic, particularly well-defined upper- and middle-crustal boundaries, results in a change in emplacement mechanism, increasing the ratio of (upper- and middle-crustal)/lower-crustal intrusions a factor of 4. This increase does not necessarily impact deep fractional crystallisation of basic melts to produce anorthosite (commonly assumed to take place at the crust–mantle boundary), but may facilitate transport through a melt-weakened hot crust. The Phanerozoic is likewise 1.5 times greater than the Proterozoic, but again this is largely a factor of decreasing crust–mantle boundary emplacement, and increasing eruption ratios of lower density mafic melts.

**S5 Calculation of intrusive-extrusive ratio for Gawler Range Volcanics and Mt Isa**

*Mt. Isa*

Mt Isa volcanics have been mapped extensively on the surface, and deep seismic profiling has allowed for the reconstruction of middle- and lower-crustal magmatic rocks emplaced at depth. We have digitised the deep seismic cross sections of MacCready (2006) and Drummond et al. (1998).

We have calculated the areas of the subsequent polygons (code provided in the repository), and divide the extrusives (E) by either the intrusives (I) or total volcanic and magmatic volume (T) to determine the E/I ratio and the E/T ratio (Figure 5). Note it is unclear whether or how much the total deeper crustal intrusives are related to the Mt Isa volcanics, as they have not been directly sampled. To account for this uncertainty, we assume upper and lower bounds of 30–60% of the volume was related to the eruptive events in calculating E/I ratios.


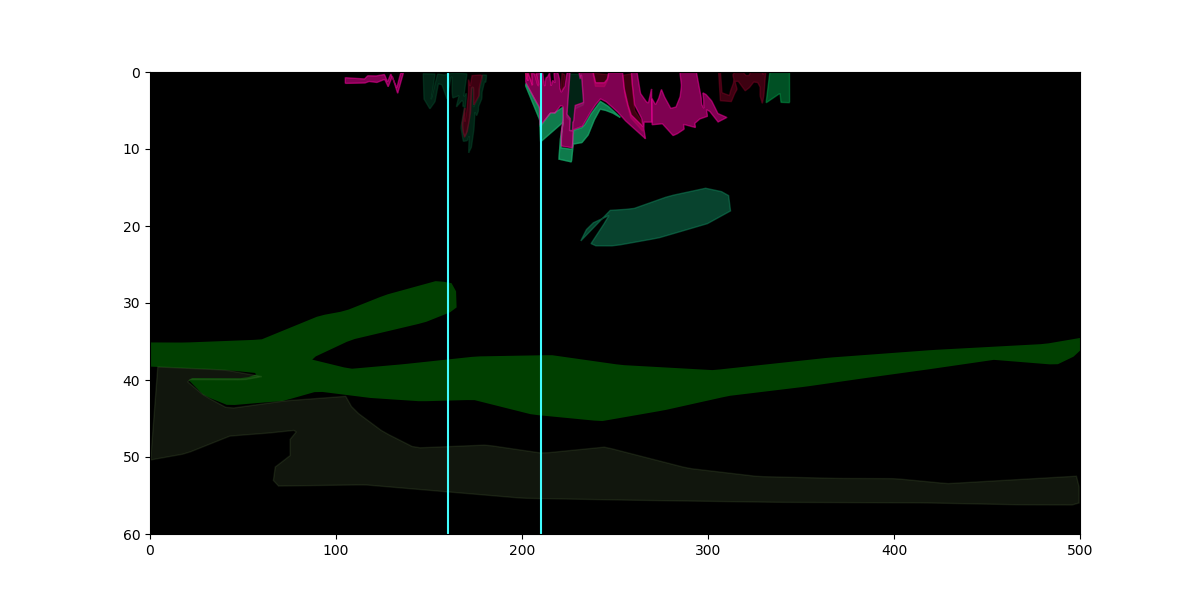


Figure S4 Major volcanic and magmatic units constrained by seismic profiling of the Mt Isa area, based on the models of MacCready (2006) and Drummond et al. (1998). Red-magenta colours represent volcanic units, and green/dark green colours represent units that were emplaced intrusively. The vertical cyan lines represent the demarcation of the Western Fold Belt (/Georgina Basin, left), Kalkadoon Leichhardt Belt (centre), and the Eastern Fold Belt (/Eromanga Basin, right). Y-axis is depth in km, x-axis is distance in km.

*Gawler Range Volcanics*

The Gawler Range volcanics are characterised by extensive lateral extrusive volcanics, and associated with a deep mafic body which is mostly constrained by its large Bouguer gravity signal.

We have obtained and modelled high-resolution gravity data from the Geoscience Australia product *Onshore geodetic Spherical Cap Bouguer gravity anomalies.* The data was downloaded from <https://data.gov.au/data/dataset/90c0e7ac-587b-45d7-adfd-931e53ba5ead>


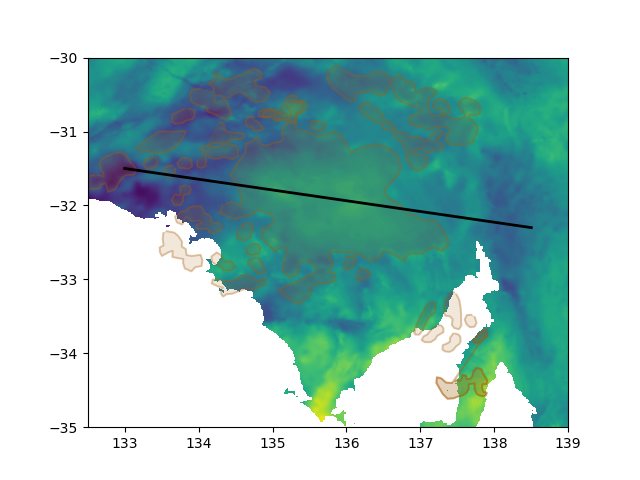


Figure S5 Gravity data over the Gawler Range Volcanics, South Australia. The data is from Geoscience Australia's National Gravity map (downloaded from <https://data.gov.au/data/dataset/90c0e7ac-587b-45d7-adfd-931e53ba5ead>). Extent of the GRVs are shown in by filled polygons (outlines from Betts et al., 2007 - includes A-type magmas of the Hiltaba suite, between 1600-1500 Ma). Dark line shows gravity transect modelled below.


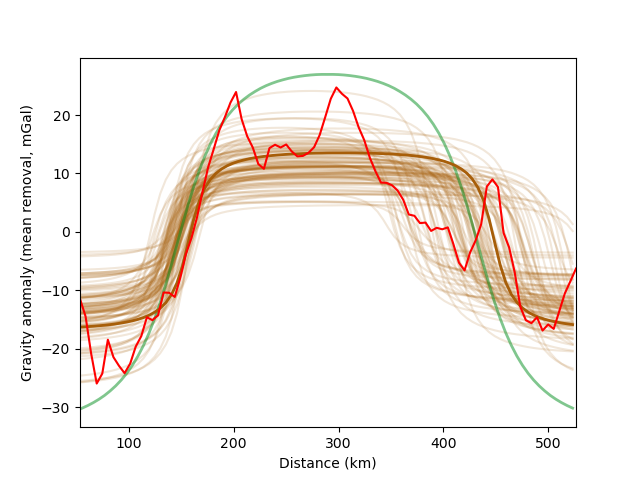


Figure S6 Gravity model over the GRV (red), transect shown in Figure S5. Also shown are the results from over 100 Monte-Carlo models for an intruded tesseroid (Uieda et al., 2016, umber coloured) with variable geometry, depth, thickness and density. Optimal model shown in thick umber line. The full implementation is in the repository script data. Green line shows the effect of emplacement of a dense anomaly at 35-40 km depth, with a density contrast appropriate to the mantle (ie. 300 kg/m3 contrast). Emplacement at the Moho results in smoother long-wavelength anomalies, and whilst they can approximate the magnitude and first-order wavelength of the GRV gravity anomaly, they cannot replicate its smaller-scale features or sharper edges of the anomaly, which requires mid-shallow crustal emplacement.

The ratio of the observed extrusive volcanics (60,000-90,000 km^3^, see Campbell (1990)), to the calculated best fit tesseroid volume for a mafic intrusion to fit the gravity data (690,717 km^3^) gives an E/I of 0.087 - 0.130, or an E/T of 0.08 - 0.115.

**S6 Anorthite trends in time**

In massif-type anorthosites, the anorthite content of plagioclase (here on referred to as An%) can vary greatly or be relatively well constrained (Ashwal and Bybee, 2017). Much of this variation can be explain as the result of fractional crystallization, polybaric crystallization and assimilation processes. However, there is a clear, steady decline in An% content over time (Fig. 1a) that is not readily explained by these processes and reflects secular change in the crust and upper mantle (Ashwal and Bybee, 2017). To understand how secular change in the crust or mantle may result in the trend observed in Figure. 1a, we must consider the variables that control anorthite content. Experimental studies of plagioclase compositions in broadly basaltic melts have highlighted important factors that control An% in crystallizing plagioclase. For example, low pressures of 3–5 kbar and high water content nearing saturation in the melt can drive plagioclase compositions to high An% (>88) (Housh and Luhr, 1991; Takagi et al., 2005). Anorthite content is correlated with higher Na/Ca and Si/Al ratios where parental magmas elevated in Na and Si favor the crystallization of plagioclase with more intermediate compositions (Panjasawatwong et al., 1995; Duncan and Green, 1987; Beard and Borgia, 1989). Temperature also controls An% as higher crystallization temperatures and faster cooling times are generally correlated with higher An% (Henry et al., 1982, Mollo et al., 2011).


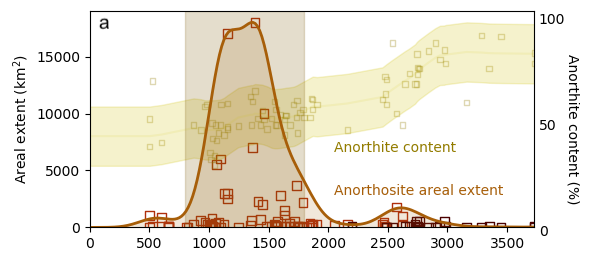


Figure S7. Anorthosite areal extent (left axis), and anorthite content of anorthosites (right axis) through time. The evolution of plagioclase composition (as anorthite content) in these anorthosites through time is calculated using a LOWESS mean fit through the data, +/- 1 STD (yellow region). Anorthite content may vary with source temperature and cooling rate, higher pressure in relation to crustal thickening, or evolution in the composition of the lower crust towards higher Na and Si (Longhi et al., 1999).

The change in anorthite content from the high An% (>80) megacrystic Archean anorthosites to more intermediate An% (40-65) in massif-type Proterozoic anorthosites results from fundamentally differing styles of magmatism related to the presence of continental crust. Archean anorthosites are associated with mafic rocks in greenstone belts and likely represent crystal accumulations in shallow, sub-volcanic magma chambers in oceanic crust (Ashwal and Bybee, 2017). Here, lower pressures and higher water content of the parental magma resulted in the crystallization of high An% (>80) plagioclase (Ashwal and Bybee, 2017). In comparison, massif-type anorthosites formed in deep seated magma chambers at the base of continental crust, fed by basaltic melts derived from the mantle or melting of the lower crust, where higher pressures favored the crystallization of plagioclase with intermediate An compositions (Ashwal and Bybee, 2017; Bybee et al., 2014).

Whilst the change in An% content across Archean anorthosites to massif-type anorthites can be reconciled by the presence or absence of continental crust, why this trend continues through the Proterozoic remains unknown. Decreasing An% may result from plagioclase crystallizing at increasingly higher pressures in relation to crustal thickening, however studies using orthopyroxene megacrysts as geobarometers do not show systematic changes in crystallization pressures in younger anorthosites compared to older occurrences (Emslie, 1975; Emslie, 1985; Charlier et al., 2010; Heinonen et al., 2020). Whilst some debate remains on the composition of the parental magmas of massif-type anorthosites, most authors agree that the mineral assemblages observed indicate a dry composition and it is unlikely that the trend results from decreasing water content in the parental magmas over time (Fram and Longhi, 1992; Longhi et al., 1999; Mitchell et al., 1995). Assimilation of lower crustal material is recognized as an important process in producing massif-type anorthosites and it maybe that the composition of the lower crust has evolved toward higher Na and Si compositions thereby progressively driving plagioclase compositions to more intermediate An% (Ashwal and Bybee, 2017; Bybee and Ashwal, 2015). Secular cooling of the mantle and crust also contributed to the decrease in An% by influencing crystallization temperatures and cooling rates.

**S7 Passive margin distribution data**

The passive margin distribution used herein is taken from Bradley (2008). In that work, he notes the collation and assessment issues with such a database. Most of the data came from accessible literature sources, including constraints on the ages of the margin. The important data for our analysis herein are the age constraints on the beginning and end of the passive margin lifetime. The uncertainties include a lack of geochronological age constraints, and difficulties in tectonic interpretation.

The onset of most passive margins in the Ancient-margins dataset of Bradley (2008) are marked by the rift–drift transition, characterised by a facies change to either platform carbonates or siliciclastics. The issues in dating this transition are outlined in Bradley (2008), and include protracted rift phases, and a lack of igneous rocks in many passive margin sequences.

Most passive margins in the Bradley database end with collision and subduction of the ocean–continent boundary, which can be dated by ashfall tuffs in the changing sedimentary sequence, or metamorphism in the orogen itself.

The data are initially separated into 3 groups, based on whether the passive margins have beginning and end age brackets (Group 1), only one bracketing age (Group 2) or no real age constraints (Group 3). We only use group 1 passive margins in our analysis.

The quality of the age constraints available are classified using the designations A, B, C or D (from best to worst). In the case of D, one or more age brackets on the margin (i.e. beginning or end) are unknown, and thus the lifetime is undetermined (these represent Group 2 and 3 above). These data were not used herein. For the highest quality ‘A’ data, we note that all examples bar one are from the Phanerozoic–Neoproterozoic, highlighting the problem of preservation and reworking in accurately identifying older margins. Most of the Mesoproterozoic examples are classified as C, with one A and nine B’s (mostly from North America) from the Mesoproterozoic–Paleoproterozoic. Largely, the classification of C for the long-lived Mesoproterozoic passive margins follows the difficulty in reconstructing tectonic controls at this time. However, there are 8 examples throughout the period 800-1800 Ma (2 B’s, 6 C’s). Six of these have lifespans greater than 370 Myrs, and together these represent a strong argument for long lifespans during this interval.

Selection and preservation issues with the margins used herein include: i) many margins may have been destroyed, and are not represented in the database; ii) a margin may be locally isolated from the global plate circuit, and not representative; iii) episodes of rapid plate activity tend to create more passive margins, but these will be on average more short-lived, iv) episodes of slow plate tectonics will generally be expected to produce *few* passive margins over a given time interval, though those that exist will be long-lived.

**Supplementary References**

Ashwal, L.D. and G.M. Bybee, *Crustal evolution and the temporality of anorthosites.* Earth-Science Reviews, 2017. **173**: p. 307-330.

Beard, J.S. and A. Borgia, *Temporal variation of mineralogy and petrology in cognate gabbroic enclaves at Arenal volcano, Costa Rica.* Contributions to Mineralogy and Petrology, 1989. **103**(1): p. 110-122.

Bybee, G.M. and L.D. Ashwal, *Isotopic disequilibrium and lower crustal contamination in slowly ascending magmas: Insights from Proterozoic anorthosites.* Geochimica et Cosmochimica Acta, 2015. **167**: p. 286-300.

Bybee, G.M., et al., *Pyroxene megacrysts in Proterozoic anorthosites: Implications for tectonic setting, magma source and magmatic processes at the Moho.* Earth and Planetary Science Letters, 2014. **389**: p. 74-85.

Campbell, E.M., 1990. *Structure and surface in the Gawler Ranges, South Australia/by Elizabeth M. Campbell* (Doctoral dissertation), University of Adelaide.

Charlier, B., et al., *Polybaric Fractional Crystallization of High-alumina Basalt Parental Magmas in the Egersund-Ogna Massif-type Anorthosite (Rogaland, SW Norway) Constrained by Plagioclase and High-alumina Orthopyroxene Megacrysts.* Journal of Petrology, 2010. **51**(12): p. 2515-2546.

Duncan, R. and D. Green, *The genesis of refractory melts in the formation of oceanic crust.* Contributions to Mineralogy and Petrology, 1987. **96**(3): p. 326-342.

Drummond, B.J., Goleby, B.R., Goncharov, A.G., Wyborn, L.A.I., Collins, C.D.N. and MacCready, T., (1998). Crustal-scale structures in the Proterozoic Mount Isa Inlier of north Australia: their seismic response and influence on mineralisation. *Tectonophysics*, *288*(1-4), pp.43-56.

Emslie, R.F., *PYROXENE MEGACRYSTS FROM ANORTHOSITIC ROCKS: NEW CLUES TO THE SOURCES AND EVOLUTION OF THE PARENT MAGMAS.* Canadian Mineralogist, 1975. **13**: p. 138-145.

Emslie, R.F., *Proterozoic Anorthosite Massifs*, in *The Deep Proterozoic Crust in the North Atlantic Provinces*, A.C. Tobi and J.L.R. Touret, Editors. 1985, Springer Netherlands: Dordrecht. p. 39-60.

Fram, M.S. and J. Longhi, *Phase equilibria of dikes associated with Proterozoic anorthosite complexes.* American Mineralogist, 1992. **77**(5-6): p. 605-616.

Heinonen, A., H. Kivisaari, and R.M. Michallik, *High-aluminum orthopyroxene megacrysts (HAOM) in the Ahvenisto complex, SE Finland, and the polybaric crystallization of massif-type anorthosites.* Contributions to Mineralogy and Petrology, 2020. **175**(1): p. 10.

Henry, D., A. Navrotsky, and H. Zimmermann, *Thermodynamics of plagioclase-melt equilibria in the system albite-anorthite-diopside.* Geochimica et Cosmochimica Acta, 1982. **46**(3): p. 381-391.

Housh, T.B. and J.F. Luhr, *Plagioclase-melt equilibria in hydrous systems.* American Mineralogist, 1991. **76**(3-4): p. 477-492.

Laske, G., Masters., G., Ma, Z. and Pasyanos, M., Update on CRUST1.0 - A 1-degree Global Model of Earth's Crust, Geophys. Res. Abstracts, 15, Abstract EGU2013-2658, 2013)

Longhi, J., et al., *Some Phase Equilibrium Constraints on the Origin of Proterozoic (Massif) Anorthosites and Related Rocks.* Journal of Petrology, 1999. **40**(2): p. 339-362.

MacCready, T., (2006) Structural cross-section based on the Mt Isa deep seismic transect, Australian Journal of Earth Sciences, 53:1, 5-26, DOI: 10.1080/08120090500431415

Mitchell, J.N., J.S. Scoates, and C.D. Frost, *High-Al gabbros in the Laramie Anorthosite Complex, Wyoming: implications for the composition of melts parental to Proterozoic anorthosite.* Contributions to Mineralogy and Petrology, 1995. **119**(2): p. 166-180.

Mollo, S., et al., *Plagioclase–melt (dis) equilibrium due to cooling dynamics: implications for thermometry, barometry and hygrometry.* Lithos, 2011. **125**(1-2): p. 221-235.

Panjasawatwong, Y., et al., *An experimental study of the effects of melt composition on plagioclase-melt equilibria at 5 and 10 kbar: implications for the origin of magmatic high-An plagioclase.* Contributions to Mineralogy and Petrology, 1995. **118**(4): p. 420-432.

Takagi, D., H. Sato, and M. Nakagawa, *Experimental study of a low-alkali tholeiite at 1–5 kbar: optimal condition for the crystallization of high-An plagioclase in hydrous arc tholeiite.* Contributions to Mineralogy and Petrology, 2005. **149**(5): p. 527-540.

Uieda, L., Barbosa, V.C. and Braitenberg, C., 2016. Tesseroids: Forward-modeling gravitational fields in spherical coordinates. *Geophysics*, *81*(5), pp.F41-F48.
